# Where Did All the Sport Go? Negative Impact of COVID-19 Lockdown on Life-Spheres and Mental Health of Spanish Young Athletes

**DOI:** 10.3389/fpsyg.2020.611872

**Published:** 2020-12-07

**Authors:** Juan Pons, Yago Ramis, Saul Alcaraz, Anna Jordana, Marta Borrueco, Miquel Torregrossa

**Affiliations:** ^1^Departament de Psicologia Bàsica, Evolutiva i de l’Educació, Facultat de Psicologia, Universitat Autònoma de Barcelona, Bellaterra, Spain; ^2^Sport Research Institute UAB, Universitat Autònoma de Barcelona, Bellaterra, Spain

**Keywords:** SARS-CoV-2, student-athletes, holistic approach, adolescent, cluster analysis, wellbeing

## Abstract

During the 2020, the pandemic caused by the massive spread of the SARS-CoV-2 coronavirus (COVID-19) resulted in a global crisis. In Spain, the COVID-19 pandemic caused a lockdown for almost 100 days and forced the sudden stop of sport practices and competitions. This interruption had a negative impact on high-level athletes’ mental health. However, its impact on young athletes, who are intrinsically developing a high-demanding dual career, remains unclear. Therefore, this study aimed at (1) describing and characterizing the general impact that COVID-19 lockdown had on Spanish young athletes’ life-spheres and mental health, and (2) identifying different profiles of athletes regarding life-conditions and sport-related variables. A sample of 544 young athletes (*M* = 15.9; *SD* = 1.51) participated in this study. Measures included life-conditions and sport-related information along with the Holistic Monitoring Questionnaire (HMQ) and the General Health Questionnaire (GHQ-12). After the screening and description of the data, profiles were defined using a two-level cluster analysis using HMQ and GHQ-12 subscales. We explored differences in demographic and sports information between profiles using MANOVA and subsequent ANOVA. Results suggest a general negative impact of COVID-19 on young athletes’ life-spheres and mental health, but with three different clusters regarding the degree of such impact. Cluster 1 grouped the 54.78% of the sample and exhibited a low negative impact of COVID-19 lockdown on life-spheres and few mental health issues. Cluster 2 grouped a 29.96% of the participants who reported a medium negative impact on life-spheres and moderate mental health issues. Cluster 3 represented 15.26% of the sample including participants who showed a high negative impact of the COVID-19 lockdown with high mental health issues. The paradigmatic participant in this third group would be a female student-athlete from a medium or low socioeconomic status with high academic demands and poor or inexistent training conditions during lockdown. Current findings emphasize the need to pay attention to young athletes’ mental health and suggest possible influencing contextual variables. We suggest some applied recommendations aimed at helping clubs and sports institutions to mitigate the negative effects of such difficult circumstances on athletes’ mental health.

## Introduction

The year 2020 will remain in History as the year of the coronavirus (COVID-19) outbreak. The COVID-19 pandemic^1^ caused a global crisis with social, political, and economic consequences. In Spain, one of the countries where the pandemic hit harder,^2^ a state of alarm was declared on March 14, letting the population on lockdown and with most of the regular activities restricted for almost 100 days. Young athletes remained without practices and competitions for months.

According to the experts in the field, mobility restrictions and social isolation policies developed during COVID-19 lockdown could have a negative impact on population mental health (e.g., Brooks et al., 2020; Papaioannou et al., 2020). Early evidence seems to confirm these predictions (Chen et al., 2020). In Spain, González-Sanguino et al. (2020) observed that between 15.8 and 21.6% of the general population showed depressive, anxious, and/or post-traumatic symptoms during the first month of confinement. However, to complement these general results, there is a need to understand better the consequences of the COVID-19 pandemic within the sports context.

Research addressing the impact of this pandemic in sports identified specific challenges for athletes’ mental health during the lockdown, such as the difficulty in keeping training conditions (Pillay et al., 2020), the social distancing from teammates (Graupensperger et al., 2020), and the uncertainty regarding the delay or cancelation of future competitions (Samuel et al., 2020). Despite most of these challenges are common for athletes of all ages and levels, research has mainly focused on high performance, professional, and/or Olympic/Paralympic athletes (see also Schinke et al., 2020a,b; Stambulova et al., 2020), letting youth sports’ reality underexplored.

Youth sport is characterized by youngsters intrinsically developing a dual career (i.e., academic education and sport combination) that requires an adequate balance of sports, academic, psychological, psychosocial, financial, and legal challenges (Wylleman, 2019). This is considered a high demanding stage that requires both high personal competencies (Miró et al., 2018; Perez-Rivases et al., 2020) and a supportive environment (Morris et al., 2020). Previous research has warned about the negative consequences that a non-successful dual career might have for athletes’ mental health (Sallen et al., 2018; Sorkkila et al., 2020), highlighting the need to approach this by taking a holistic approach (e.g., Wylleman and Lavallee, 2004; Wylleman, 2019) that captures both indirect (e.g., impact on life-spheres) and direct (e.g., anxiety/depression and social dysfunction) indicators of athletes’ mental health.

Considering the changes that COVID-19 lockdown meant for young athletes’ dual career (i.e., new academic reality, practices and competitions canceled, and social distance from peers), and the lack of resources in certain athletic environments for youngsters developing a dual career in Spain (Mejías et al., 2020), it is needed to understand the complexity of the recent confinement in youth sport. Concretely, the aim of this study was two-fold: (1) to describe and characterize the general impact that COVID-19 lockdown had on Spanish young athletes’ life-spheres and mental health and (2) to identify different profiles of athletes regarding these target variables along with their life-conditions and sport-related characteristics.

## Materials and Methods

### Participants

A total of 833 athletes participated in this study. After applying the inclusion (i.e., having between 13 and 18 years) and exclusion (i.e., not completing the full survey or completing it in less than 3 min) criteria, the final sample consisted of 544 athletes (*M*_age_ = 15.9; *SD* = 1.51; ♀ = 48.5%). Participants were lower secondary school (63.2%), upper secondary school (27.8%), or tertiary education students (9.0%). The sample was composed of athletes having from 1 to 15 years of sports experience (*M*_experience_ = 7.11; *SD* = 3.11), competing at regional (38.1%), autonomic (32.5%), national (23.0%), or international level (6.4%). Most athletes reported having an average (58.6%) or good (32.2%) socioeconomic level and living in a home (50.6%) or flat (33.5%) with garden or courtyard. Nearly all participants (98.9%) stayed with their families during the COVID-19 lockdown. When answering the questionnaire, all of them had accumulated between 50 and 100 days of confinement (*M*_days_ = 72.71; *SD* = 12.03).

### Measures

#### Demographic and Sports Information

An *ad hoc* survey was created to assess relevant information for the objectives of this study. This survey was composed of items evaluating (a) life-conditions during COVID-19 lockdown (i.e., perceived socioeconomic level, academic course, number of co-habitants at home, and physical activity levels) and sport-related characteristics (i.e., years of sporting experience, competitive level, training conditions, and perceived support by the club and/or sports institutions).

#### Negative Impact of COVID-19 Lockdown on Life-Spheres

Impact of lockdown on athletes’ life-domains was assessed using the Holistic Monitoring Questionnaire (HMQ; De Brandt et al., 2019). This questionnaire contains 11 items that evaluate the perceived impact of certain events, in this case the COVID-19 lockdown, on athletes’ different life-spheres (e.g., “Please, rate the impact that the COVID-19 situation is having on your different life domains”). The HMQ is divided into four different life-spheres: (1) Dual career (i.e., studies, sport, social life, and the combination of these three areas); (2) Health (i.e., physical health, mental health, mood, and well-being); (3) Rest and recovery (i.e., sleep quality and recovery from training sessions); and (4) Economy (i.e., financial situation). Due to the characteristics of the participants of this study (i.e., economic dependence from their parents), the financial situation item was not included. All items were rated on a 5-point Likert-style scale from 1 (*very positive impact*) to 5 (*very negative impact*). Cronbach’s alpha coefficient showed acceptable-to-good reliability indicators for dual career (*α* = 0.74), health (*α* = 0.81), and rest and recovery (*α* = 0.85) subscales. Confirmatory factor analysis showed acceptable model fit indicators, with *χ*^2^(32) = 203.55, *p* < 0.001, RMSEA = 0.098, 90% CI (0.085, 0.111), CFI = 0.973, and TLI = 0.961.

#### Mental Health Issues

We used the Spanish version of the General Health Questionnaire-12 (GHQ-12; Goldberg et al., 1997; Sánchez-López and Dresch, 2008). This 12-item self-administered questionnaire screens different symptoms that are indicative of a poor mental health. Respondents must select the option that better fits their degree of agreement in a 7-point Likert scale from 1 (*strongly disagree*) to 7 (*strongly agree*). Following past research with similar samples (e.g., Padrón et al., 2012), items were grouped into (1) anxiety and depression, (2) social dysfunction, and (3) loss of confidence subscales. We reworded the stem of the questionnaire to adapt it to the aims of this study (i.e., “during last few weeks under the situation of confinement…”). Cronbach’s alpha coefficient reported acceptable levels of reliability, with values of 0.79, 0.76, and 0.80, respectively. Confirmatory factor analysis presented acceptable model fit indices, with *χ*^2^(51) = 309.06, *p* < 0.001, RMSEA = 0.096, 90% CI (0.086, 0.107), CFI = 0.949, and TLI = 0.933.

### Procedure

After obtaining the approval of the University ethics committee (reference 4996), we designed an online tool with the aforementioned questionnaires using LimeSurvey software. A total of 30 sport clubs and sports institutions with different sports characteristics (i.e., number of athletes, competition level, and sport type) were invited to participate in this study using a convenience sampling strategy. Twenty-six of them agreed to participate. We did not detect any common pattern between entities that declined to participate in the study. For those entities that agreed to participate, we asked them to share with their athletes the link to the survey and some relevant information about the research (e.g., invitation letter, survey instructions, and contact information). All participants signed an informed consent and were informed about the confidential and voluntary nature of the study and the absence of any kind of reward to participate. As a gratitude for their help, a global report with the main findings of the study was sent to that all entities that participated in the data collection.

### Data Analysis

All analyses were conducted using SPSS 19.0 statistical package. Preliminary data analysis included the examination of the missing data patterns and multivariate outliers. Listwise deletion method was used to deal with missing data. Accordingly, partial responses were not considered in this study. Cases with responses outside the three interquartile ratio thresholds were also deleted (Hoaglin and Iglewicz, 1987). Afterwards, we calculated the descriptive statistics of the whole dataset, including means, standard deviations, bivariate correlations using Pearson’s correlation coefficient, and reliability using Cronbach’s alpha coefficient. Such descriptive analyses provided an overall understanding of Spanish young athletes during lockdown.

Next, we used the cluster analysis technique to characterize the different athletes depending on the impact that COVID-19 lockdown had on them. This characterization was conducted following the next steps: (1) we selected subscales of HMQ and GHQ-12 as clustering variables. Then, (2) we transformed the clustering variables into *z*-scores and (3) we combined both hierarchical and non-hierarchical cluster analysis in order to obtain a more stable solution (see Hair et al., 2010). Hierarchical cluster analysis was calculated using Ward’s linkage and squared Euclidean distance. The number of clusters was derived from the visual inspection of the dendrogram and the Bayesian and Akaike Information Criteria (BIC and AIC; Akaike, 1974). The resulting cluster centers were used as starting points for the *k*-means cluster analysis, which tests the adequacy of a specified cluster solution. Such analyses identified different profiles of young athletes regarding the impact that COVID-19 had on their life.

Last, to approach the second aim of the study, we compared different life-conditions and sport-related variables between the resulting clusters. For categorical data, we used Pearson’s Chi-square test and Fisher’s exact test (when contingency tables contained at least a cell with less than five cases) with Monte Carlo iteration method. This test was computed considering the need to correct the value of *p* when conducting multiple comparisons (*p* = 0.017). For numeric data, we first used MANOVA to test whether compared groups differed in the explored dependent variables. Subsequently, we conducted ANOVA with *post-hoc* test comparisons using Games-Howell procedure to evaluate the differences between clusters in life-conditions and sport-related variables.

## Results

### Descriptive Statistics

Descriptive statistics are presented in Table 1. Overall, results indicate that athletes reported that COVID-19 lockdown had a negative impact on their life-spheres and mental health. Regarding the different life-spheres, this impact seems to be more negative on dual career (*M* = 3.6; *SD* = 0.7; Rank = 1–5) and health domains (*M* = 3.3; *SD* = 0.7; Rank = 1–5) than in the rest and recovery domain (*M* = 2.9; *SD* = 1.0; Rank = 1–5). For mental health issues, young athletes reported, on average, higher anxious/depressive (*M* = 3.5; *SD* = 1.0; Rank = 1–7) and social dysfunction (*M* = 3.5; *SD* = 1.5; Rank = 1–7) symptoms in comparison with loss of confidence (*M* = 2.6; *SD* = 1.7; Rank = 1–7). Descriptive results also indicate positive correlations between life-spheres and mental health, with ranging magnitudes from 0.13 to 0.59 among them. Notably, social dysfunction showed to be the most correlated factor with the negative impact on life-spheres subscales (dual career: *r* = 0.43, health: *r* = 0.58, rest and recovery: *r* = 0.26).

**Table 1:** Descriptive statistics of negative impact of COVID-19 on life-spheres and mental health issues.

	*M*	*SD*	Range	*α*	1	2	3	4	5	6
***Negative impact of COVID-19 lockdown on life-spheres***										
1. Dual career	3.59	0.74	1–5	0.74	-					
2. Health	3.27	0.68	1–5	0.81	0.633^*^	-				
3. Rest and recovery	2.92	0.99	1–5	0.85	0.204^*^	0.364^*^	-			
***Mental health issues***										
4. Anxiety and depression	3.46	1.04	1–7	0.79	0.180^*^	0.418^*^	0.273^*^	-		
5. Social dysfunction	3.53	1.50	1–7	0.76	0.428^*^	0.580^*^	0.263^*^	0.492^*^	-	
6. Loss of confidence	2.61	1.67	1–7	0.80	0.128^*^	0.375^*^	0.209^*^	0.600^*^	0.509^*^	-

Bivariate correlations were computed using Pearson’s coefficient.

* *p* < 0.001.

### Cluster Analysis

**Figure 1:**
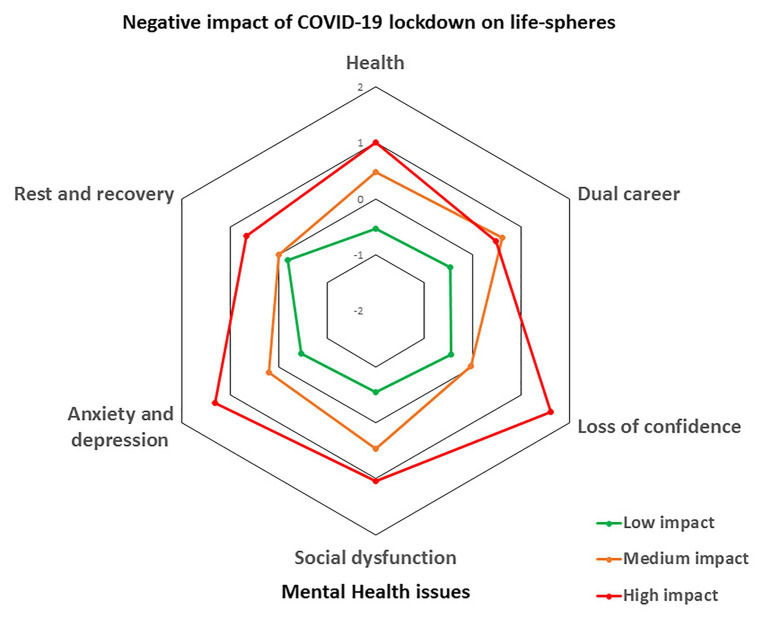
Accepted cluster solution (standardized scores).

Results from the hierarchical cluster analysis suggested the three-cluster solution as the most suitable (see Figure 1 and Table 2). This structure was subsequently confirmed by the non-hierarchical *k*-means analysis and was considered as theoretically satisfactory. The MANOVA suggested the existence of significant differences between groups in all clustering variables (Pillai’s trace = 0.95; *p* < 0.001). The resulting profiles were labeled as *low impact*, *medium impact*, and *high impact* clusters. Overall, these three profiles present differences in the way that COVID-19 lockdown impacted on athletes’ life-spheres and mental health. For most of the participants, the impact was low or medium, but there was also a considerable group of athletes that reported important negative consequences. Concretely, the 54.78% of the sample (*n* = 298) belong to the *low impact* cluster, the 29.96% of the sample (*n* = 163) to the *medium impact* cluster, and the 15.26% of the sample (*n* = 83) to the *high-impact* cluster.

**Table 2:** Standardized and non-standardized scores in the clustering variables of the accepted cluster solution.

	Cluster 1 (*n* = 298): *low impact*		Cluster 2 (*n* = 163): *medium impact*		Cluster 3 (*n* = 83): *high impact*		*F*	*p*
	*Z*-scores	Raw scores	*Z*-scores	Raw scores	*Z*-scores	Raw scores		
***Negative impact of COVID-19 lockdown on life-spheres***								
Dual career	−0.47 (0.06)^c2c3^	3.25 (0.04)	0.61 (0.04)^c1^	4.04 (0.03)	0.48 (0.08)^c1^	3.95 (0.06)	97.93	**<0.001**
Health	−0.54 (0.05)^c2c3^	2.90 (0.04)	0.48 (0.05)^c1c3^	3.60 (0.03)	1.01 (0.07)^c1c2^	3.96 (0.05)	171.00	**<0.001**
Rest and recovery	−0.18 (0.05)^c3^	2.74 (0.05)	0.00 (0.09)^c3^	2.92 (0.08)	0.67 (0.11)^c1c2^	3.58 (0.11)	25.66	**<0.001**
***Mental health issues***								
Anxiety and depression	−0.47 (0.05)^c2c3^	2.82 (0.08)	0.20 (0.05)^c1c3^	3.83 (0.08)	1.31 (0.06)^c1c2^	5.50 (0.09)	179.55	**<0.001**
Social dysfunction	−0.55 (0.04)^c2c3^	2.89 (0.04)	0.47 (0.06)^c1c3^	3.95 (0.07)	1.04 (0.10)^c1c2^	4.55 (0.10)	176.08	**<0.001**
Loss of confidence	−0.44 (0.04)^c2c3^	1.88 (0.07)	−0.03 (0.06)^c1c3^	2.56 (0.09)	1.62 (0.07)^c1c2^	5.31 (0.12)	276.79	**<0.001**

C1, Cluster 1; C2, Cluster 2; C3, Cluster 3. Boldface indicates inter-group significant differences. Superscripts indicate intra-group significant differences using Games-Howell *post-hoc* test.

The *low impact* cluster included those athletes who reported a small negative impact of COVID-19 on life-spheres (dual career: *Z* = −0.47, health: *Z* = −0.54, rest and recovery: *Z* = −0.18) and few mental health issues (anxiety and depression: *Z* = −0.47, social dysfunction: *Z* = −0.55, loss of confidence: *Z* = −0.44). Second, the *medium impact* cluster contained those athletes who reported a medium negative impact of COVID-19 lockdown on life-spheres (dual career: *Z* = 0.61, health: *Z* = 0.48; rest and recovery: *Z* = 0) and moderate mental health issues (anxiety and depression: *Z* = 0.20, social dysfunction: *Z* = 0.47, loss of confidence: *Z* = −0.03). Finally, the *high impact* cluster included those athletes who reported a considerable negative impact of COVID-19 lockdown on life-spheres (dual career: *Z* = 0.48, health: *Z* = 1.01; rest and recovery: *Z* = 0.67) and high mental health issues (anxiety and depression *Z* = 1.31, social dysfunction *Z* = 1.04, loss of confidence *Z* = 1.62).

### Differences Between Clusters

We compared the groups derived from the cluster analysis considering life-conditions and sports-related information to further characterize each observed profile (see Tables 3A and 3B). The three clusters presented no differences regarding the number of cohabitants during lockdown, physical activity levels before the pandemic, years of sports experience, and competitive level. However, these three groups differed in terms of gender, socioeconomic level, academic course, physical activity and training conditions during lockdown, and perceived support from sport clubs or institutions (*p* < 0.05). *Post-hoc* comparisons between groups showed that, compared to *low impact* and *medium impact* clusters, athletes in the *high impact* cluster were predominantly (72.0%) women (*p*_1–3_ < 0.001, *p*_2–3_ = 0.005) reporting a lower socioeconomic level (*p*_1–3_ = 0.001, *p*_2–3_ = 0.006), a higher academic course (*p*_1–3_ < 0.001, *p*_2–3_ = 0.057), and worse training conditions under lockdown (*p*_1–3_ = 0.008, *p*_2–3_ = 0.098). As illustrated in Tables 3A and 3B, these differences are especially evident between *low impact* and *high impact* groups.

**Table 3A:** Differences in demographic and sports information between clusters (ordinal variables).

	Cluster 1: *low impact* (*n* = 298)	Cluster 2: *medium impact* (*n* = 163)	Cluster 3: *high impact* (*n* = 83)	*χ*^2^ (gl)^f^	*p*
*Gender*	C3	C3	C1 C2	26.70 (2)	**<0.001**
Male	59.6%	47.9%	28.0%		
Female	40.4%	52.1%	72.0%		
*Socioeconomic level*	C3	C3	C1 C2	21.02 (8)	**0.003**
Very bad	0%	0%	2.4%		
Bad	4.0%	4.3%	14.5%		
Medium	60.1%	57.7%	55.4%		
Good	31.9%	35.0%	27.7%		
Very Good	4.0%	3.1%	0%		
*Academic course*^a^	C2 C3	C1	C1	48.02 (10)	**<0.001**
1° ESO (year 8)	7.1%	5.0%	0%		
2° ESO (year 9)	23.8%	13.6%	6.8%		
3° ESO (year 10)	23.5%	20.0%	13.5%		
4° ESO (year 11)	22.8%	25.7%	29.7%		
1° BACHILLER (year 12)	10.0%	25.7%	31.1%		
2° BACHILLER (year 13)	12.8%	10.0%	18.9%		
*Competitive level*				4.37 (6)	0.634
Regional	35.2%	39.3%	45.8%		
Autonomic	35.2%	29.4%	28.9%		
National	22.5%	25.2%	20.5%		
International	7.1%	6.1%	4.8%		
*Lockdown training conditions*	C3		C1	12.63 (6)	**0.045**
Not training	10.6%	12.5%	24.7%		
Training with important changes	55.0%	57.5%	54.3%		
Training with minor changes	27.6%	25.0%	18.5%		
Training as always	6.8%	5.0%	2.5%		
*Satisfaction with club/institution*	C2	C1		22.87 (8)	**0.003**
Very unsatisfied	4.7%	3.1%	8.4%		
Unsatisfied	3.7%	9.3%	4.8%		
Indifferent	19.5%	22.2%	24.1%		
Satisfied	32.7%	40.1%	42.2%		
Very satisfied	39.4%	25.3%	20.5%		

C1, Cluster 1; C2, Cluster 2; C3, Cluster 3. Boldface indicates inter-group significant differences (*p* < 0.05). Superscripts indicate intra-group significant differences (corrected *p* < 0.017) using Chi-square test.

a Academic course was computed removing those participants pursuing tertiary education programs (participants excluded = 49).

f All inter-group comparisons that included a group with *n* < 5, were computed using Fishers’ exact test.

**Table 3B:** Differences in demographic and sports information between clusters (numerical variables).

	Cluster 1 (*n* = 298): low impact		Cluster 2 (*n* = 163): medium impact		Cluster 3 (*n* = 83): high impact		*F*	*p*
	*M*	*SD*	*M*	*SD*	*M*	*SD*		
Number of co-habitants	4.02	0.89	3.94	0.81	4.01	1.09	0.38	0.682
PA time before lockdown	9.45	6.02	9.31	5.22	8.08	3.91	2.05	0.130
PA time during lockdown	5.81	4.29	4.94	3.58	4.83	3.94	3.42	**0.034**
Sports experience	7.16	0.18	7.33	0.24	6.49	0.35	2.09	0.125

PA, physical activity. Boldface indicates inter-group significant differences.

## Discussion

This study aimed at better understanding the impact of confinement on young athletes by (1) describing and characterizing the general impact of COVID-19 lockdown on Spanish young athletes’ life-spheres and mental health and (2) identifying different profiles of athletes regarding these target variables along with their life-conditions and sport-related characteristics. Globally, results showed a negative impact of COVID-19 lockdown on the sample, but with three different profiles regarding the degree of such impact. Especially, the *high impact* cluster showed a substantial negative effect of confinement on young athletes’ life-spheres and mental health. In comparison with the other two groups, this cluster is mostly composed of female athletes, with a lower socioeconomic level, higher academic course, and worse training conditions under lockdown. The present results expand the previous knowledge about the COVID-19 pandemic by providing new insights on the mental health status of young athletes, a frequently underexplored population in mental health research.

Descriptive results show a negative impact of COVID-19 lockdown on athletes’ life-spheres and mental health, being the dual career factor the most negatively affected. This finding is in line with the predictions about the negative consequences of confinement (Brooks et al., 2020; Papaioannou et al., 2020), and the importance of an adequate dual career balance for mental health (Schinke et al., 2017). Similar results were found in early evidence exploring the impact of COVID-19 lockdown in similar samples (di Fronso et al., 2020; Pillay et al., 2020). However, Clemente-Suárez et al. (2020) reported no effects on Spanish Olympic athletes’ mental health during the first month of confinement. The differences between studies are probably due to the diverse characteristics of the samples (i.e., as pointed by the authors, Olympic athletes are more used to cope with challenging situations) and to the number of days of confinement when participating in each study.

Results from cluster analysis indicate differences regarding how young athletes experienced the COVID-19 lockdown, suggesting different impact realities. In line with the results found in non-sportive samples (e.g., Chen et al., 2020), most youngsters showed a low-to-medium impact of confinement. However, a substantial percentage (i.e., 15.26%) of young athletes reported a high impact of COVID-19 lockdown on their life-spheres and mental health. According to previous research, this group could be at risk for suffering from psychological problems. Considering that, adolescent athletes are a population with a low tendency for help-seeking behaviors (Tomalski et al., 2019), present results emphasize the need to pay attention to young athletes’ mental health to prevent potential psychosocial problems.

A second aim of this study was to explore the differences between clusters regarding general demographic and life-conditions and sports-related characteristics. Group comparisons showed that, compared to the other two groups, the *high impact* cluster had a greater percentage of female athletes exhibiting a lower socioeconomic level, who reported pursuing higher academic courses and having poorer training conditions during confinement. Although these differences cannot be interpreted in a causal way, present findings suggest some research lines to be explored in the future. Regarding gender, research addressing differences in how the pandemic hit male and female athletes has revealed a more negative impact of COVID-19 lockdown in female athletes (di Fronso et al., 2020). In this line, Bowes et al. (2020) identified some factors that might help to explain these differences in elite sport, such as a later resumption of the sport activity, greater difficulties for training at home, and a lesser financial support. As suggested in this study, future research in the field of youth sport should explore in more detail the sociological factors causing these gender differences beyond the impact of COVID-19 lockdown.

Group differences regarding academic course showed that participants in the *high impact* cluster were pursuing higher academic courses than the *low impact* one. Research in this line suggested that individuals with higher academic level usually report lower levels of well-being (Wanberg et al., 2020). Within the sports field, previous evidence relates mental health issues with higher difficulties to undertake a dual career pathway (Sallen et al., 2018; Sorkkila et al., 2020). As highlighted by other authors (e.g., Samuel et al., 2020; Stambulova et al., 2020), COVID-19 lockdown can be understood as a change-event with unexpected demands for the athletes that combine academic and sports career. Therefore, it would be interesting that future research addresses the impact of this adaptation process on young athletes’ mental health.

Regarding the socioeconomic level, group comparisons showed that the *high impact* cluster had a worse socioeconomic level than *medium* and *low impact* ones. While lower socioeconomic level has been identified as a risk factor associated with mental health issues (e.g., Jones et al., 2016), recent research specifically addressing the impact of the pandemic suggests that people with higher incomes experienced a greater relative decrease in well-being during lockdown (Wanberg et al., 2020). In line with this relative perception of loss, our results also showed group differences regarding training conditions during confinement, with the *high impact* cluster showing worse training conditions than the *low impact* cluster. Faulkner et al. (2020) had suggested that regular and structured physical activity would act as a coping strategy for mitigating COVID-19 lockdown negative consequences. However, further research should address whether the socioeconomic conditions of the athletic population might be interrelated with training conditions. In this line, we hypothesize that athletes with more resources would be more likely to keep similar training conditions during COVID-19 lockdown and perhaps is this perception of preservation of conditions despite the general conditions what would have a positive effect.

### Practical Implications

Current findings entail some appealing applied considerations. By presenting the way that COVID-19 impacted young athletes, this research contributes to the visibility of potential mental health issues in the youth sport population. In this sense, and in view of the observed contextual differences between profiles partly related with the backing of sport institutions, we recommend clubs and sport organizations to promote youngsters’ physical activity and to facilitate the access to supportive network during challenging circumstances even when the competitions are canceled (e.g., lockdown, high-demanding academic courses, and career transitions). Special attention should be given to women’s sport, as female athletes seem to be the least-favored group. By developing these supportive resources (e.g., dual career support services and communication networks with athletes and families) that serve as backup system for young athletes, sports clubs and institutions will become protective agents against mental health issues in this population.

### Limitations and Future Directions

Some considerations are needed when evaluating the reported results. First, it is important to bear in mind that this is a descriptive study, therefore, while group comparisons may indicate a possible role of certain life-conditions and sport-related variables in the health of young athletes, these relations cannot be interpreted in a causal way. Future studies should continue developing this approach using designs that allow making predictive or causal attributions. Second, this work is a first attempt to study the psychometric properties of the Holistic Monitoring Questionnaire (HMQ; De Brandt et al., 2019). However, more evidence is needed in different athletic samples to confirm the satisfactory properties regarding internal consistency and factorial structure obtained in this study.

As a complement of the descriptive nature of these findings, further studies should deepen some other research questions regarding the effects of the COVID-19 pandemic in this population. Specifically, focusing on reporting complementary mental health indicators (e.g., performance, well-being, and basic psychological needs satisfaction; Schinke et al., 2017) in this sample, evaluating the long-term impact of lockdown on mental health, and/or implementing intervention programs aimed at improving the mental health status of highly affected athletes could be of great interest.

### Conclusion

The COVID-19 lockdown had a general negative impact on Spanish young athletes. However, significant differences were found when comparing different individual realities. While most of the sample reported a low-to-moderate impact, an important group of participants reported high negative issues in relation to this COVID-19 lockdown. Group comparisons showed that the *high impact* cluster was composed mostly of female athletes with lower socioeconomic level, pursuing a more demanding academic course and reporting worse training conditions than their counterparts. This study reinforces the need to focus on young (specially women’s) athletes’ mental health and highlights the role of contextual variables with potential issues. Sport clubs and institutions, by supporting young athletes during difficult moments, could and should help mitigating the negative impact of such challenging circumstances on their athletes’ mental health.

## Data Availability Statement

The raw data supporting the conclusions of this article will be made available by the authors, without undue reservation.

## Ethics Statement

The studies involving human participants were reviewed and approved by *Comissió d’Ètica en l’Experimentació Animal i Humana*, Universitat Autònoma de Barcelona. Written informed consent to participate in this study was provided by the participants’ legal guardian/next of kin.

## Author Contributions

JP, YR, SA, and MT participated in the design of the research project. JP, AJ, and MB participated in the survey design and data collection. JP, YR, and SA participated in the data analysis. All authors participated in the preparation of the manuscript. All authors contributed to the article and approved the submitted version.

### Conflict of Interest

The authors declare that the research was conducted in the absence of any commercial or financial relationships that could be construed as a potential conflict of interest.

## Footnotes

^1^Similar to Stambulova et al. (2020), authors differentiate between **COVID-19** as a syndrome caused by the SARS-CoV-2, **COVID-19 pandemic** as the current global crisis triggered due to the spread of COVID-19 worldwide, and the **COVID-19 lockdown** as the period of confinement of the population that most countries adopted to stop the COVID-19 spread.

^2^https://www.worldometers.info/coronavirus/

## References

[ref1] Akaike H. (1974). A new look at the statistical model identification. IEEE Trans. Aut. Cont. 19, 716–723. 10.1109/TAC.1974.1100705

[ref2] Bowes A. Lomax L. Piasecki J. (2020). The impact of the COVID-19 lockdown on elite sportswomen. Manag. Sport Leis. 10.1080/23750472.2020.1825988 [Epub ahead of print]

[ref3] Brooks S. K. Webster R. K. Smith L. E. Woodland L. Wessely S. Greenberg N.. (2020). The psychological impact of quarantine and how to reduce it: rapid review of the evidence. Lancet 395, 912–920. 10.1016/S0140-6736(20)30460-8, PMID: 32112714 PMC7158942

[ref4] Chen B. Sun J. Feng Y. (2020). How have COVID-19 isolation policies affected young people’s mental health? Evidence from Chinese college students. Front. Psychol. 11:1529. 10.3389/fpsyg.2020.01529, PMID: 32670172 PMC7327104

[ref5] Clemente-Suárez V. J. Fuentes-García J. P. de la Vega Marcos R. Martínez Patiño M. J. (2020). Modulators of the personal and professional threat perception of Olympic athletes in the actual COVID-19 crisis. Front. Psychol. 11:1985. 10.3389/fpsyg.2020.01985, PMID: 32849157 PMC7419607

[ref6] De Brandt K. Wylleman P. De Knop P. (2019). “Optimizing student-athletes’ dual career at the Vrije Universiteit Brussel.” in *Presentation during the international symposium ‘Dual Career Day’*; Brussels, Belgium.

[ref7] di Fronso S. Costa S. Montesano C. Di Gruttola F. Ciofi E. G. Morgilli L. (2020). The effects of COVID-19 pandemic on perceived stress and psychobiosocial states in Italian athletes. Int. J. Sport Exerc. Psychol. 10.1080/1612197X.2020.1802612 [Epub ahead of print]

[ref8] Faulkner G. Rhodes R. E. Vanderloo L. M. Chulak-bozer T. Reilly N. O. Ferguson L. (2020). Physical activity as a coping strategy for mental health due to the COVID-19 virus: a potential disconnect among Canadian adults? Front. Commun. 5:571833. 10.3389/fcomm.2020.571833

[ref9] Goldberg D. P. Gater R. Sartorius N. Ustun T. B. Piccinelli M. Gureje O.. (1997). The validity of two versions of the GHQ in the WHO study of mental illness in general health care. Psychol. Med. 27, 191–197. 10.1017/S0033291796004242, PMID: 9122299

[ref10] González-Sanguino C. Ausín B. ÁngelCastellanos M. Saiz J. López-Gómez A. Ugidos C.. (2020). Mental health consequences during the initial stage of the 2020 coronavirus pandemic (COVID-19) in Spain. Brain Behav. Immun. 87, 172–176. 10.1016/j.bbi.2020.05.040, PMID: 32405150 PMC7219372

[ref11] Graupensperger S. Benson A. J. Kilmer J. R. Evans M. B. (2020). Social (un)distancing: teammate interactions, athletic identity, and mental health of student-athletes during the COVID-19 pandemic. J. Adolesc. Health 67, 662–670. 10.1016/j.jadohealth.2020.08.001 32943294 PMC7489994

[ref12] Hair J. F. Black W. C. Babin B. J. Anderson R. E. (2010). Multivariate data analysis. 7th Edn. New Jersey: Prentice Hall.

[ref13] Hoaglin D. C. Iglewicz B. (1987). Fine-tuning some resistant rules for outlier labeling. J. Am. Stat. Assoc. 82, 1147–1149. 10.1080/01621459.1987.10478551

[ref14] Jones D. J. Anton M. Zachary C. Pittman S. Turner P. Forehand R.. (2016). A review of the key considerations in mental health services research: a focus on low-income children and families. Couple Fam. Psychol. Res. Pract. 5, 240–257. 10.1037/cfp0000069, PMID: 28503361 PMC5424605

[ref15] Mejías J. Torregrossa M. Jordana A. Borrueco M. Pons J. Ramis Y. (2020). Taxonomía de entornos desarrolladores de carrera dual en España [A taxonomy of dual career development environments in Spain]. Cult. Cienc. y Deporte. 10.12800/ccd.v16i47.1624 [Epub ahead of print]

[ref16] Miró S. Pérez-Rivases A. Ramis Y. Torregrossa M. (2018). ¿Compaginar o elegir?: la transición del bachillerato a la universidad de deportistas de alto rendimiento [Balancing or choosing? The transition from high school to university of high performance athletes]. Rev. Psicol. del Deporte 27, 59–68.

[ref17] Morris R. Cartigny E. Ryba T. V. Wylleman P. Henriksen K. Torregrossa M. (2020). A taxonomy of dual career development environments in European countries. Eur. Sport Manag. Q. 10.1080/16184742.2020.1725778 [Epub ahead of print]

[ref18] Padrón A. Galań I. Durbań M. Gandarillas A. Rodríguez-Artalejo F. (2012). Confirmatory factor analysis of the General Health Questionnaire (GHQ-12) in Spanish adolescents. Qual. Life Res. 21, 1291–1298. 10.1007/s11136-011-0038-x, PMID: 21997139

[ref19] Papaioannou A. G. Schinke R. J. Chang Y. K. Kim Y. H. Duda J. L. (2020). Physical activity, health and well-being in an imposed social distanced world. Int. J. Sport Exerc. Psychol. 18:414. 10.1080/1612197X.2020.1773195

[ref20] Perez-Rivases A. Pons J. Regüela S. Viladrich C. Torregrossa M. Regüela S. (2020). Spanish female student-athletes’ perception of key competencies for successful dual career adjustment. Int. J. Sport Exerc. Psychol. 10.1080/1612197X.2020.1717575 [Epub ahead of print]

[ref21] Pillay L. Janse van Rensburg D. C. C. Jansen van Rensburg A. Ramagole D. A. Holtzhausen L. Dijkstra H. P.. (2020). Nowhere to hide: the significant impact of coronavirus disease 2019 (COVID-19) measures on elite and semi-elite South African athletes. J. Sci. Med. Sport 23, 670–679. 10.1016/j.jsams.2020.05.016, PMID: 32448749 PMC7235602

[ref22] Sallen J. Hemming K. Richartz A. (2018). Facilitating dual careers by improving resistance to chronic stress: effects of an intervention programme for elite student athletes. Eur. J. Sport Sci. 18, 112–122. 10.1080/17461391.2017.1407363, PMID: 29199550

[ref23] Samuel R. D. Tenenbaum G. Galily Y. (2020). The 2020 coronavirus pandemic as a change-event in sport performers’ careers: conceptual and applied practice considerations. Front. Psychol. 11:567966. 10.3389/fpsyg.2020.567966, PMID: 33071895 PMC7540073

[ref24] Sánchez-López M. D. P. Dresch V. (2008). The 12-item General Health Questionnaire (GHQ-12): reliability, external validity and factor structure in the Spanish population. Psicothema 20, 839–843. PMID: 18940092

[ref25] Schinke R. J. Papaioannou A. Henriksen K. Si G. Zhang L. Haberl P. (2020a). Sport psychology services to high performance athletes during COVID-19. Int. J. Sport Exerc. Psychol. 18:269. 10.1080/1612197X.2020.1754616

[ref26] Schinke R. J. Papaioannou A. Maher C. Parham W. D. Larsen C. H. Gordin R. (2020b). Sport psychology services to professional athletes: working through COVID-19. Int. J. Sport Exerc. Psychol. 18:409. 10.1080/1612197X.2020.1766182

[ref27] Schinke R. J. Stambulova N. B. Si G. Moore Z. (2017). International society of sport psychology position stand: athletes’ mental health, performance, and development. Int. J. Sport Exerc. Psychol. 16:622. 10.1080/1612197X.2017.1295557

[ref28] Sorkkila M. Ryba T. V. Selänne H. Aunola K. (2020). Development of school and sport burnout in adolescent student-athletes: a longitudinal mixed-methods study. J. Res. Adolesc. 30, 115–133. 10.1111/jora.12453, PMID: 30207416

[ref29] Stambulova N. B. Schinke R. J. Lavallee D. Wylleman P. (2020). The COVID-19 pandemic and Olympic/Paralympic athletes’ developmental challenges and possibilities in times of a global crisis-transition. Int. J. Sport Exerc. Psychol. 10.1080/1612197X.2020.1810865 [Epub ahead of print]

[ref30] Tomalski J. Clevinger K. Albert E. Jackson R. Wartalowicz K. Petrie T. A. (2019). Mental health screening for athletes: program development, implementation, and evaluation. J. Sport Psychol. Action 10:121. 10.1080/21520704.2019.1604589

[ref31] Wanberg C. R. Csillag B. Douglass R. P. Zhou L. Pollard M. S. (2020). Socioeconomic status and well-being during COVID-19: a resource-based examination. J. Appl. Psychol. 10.1037/apl0000831, PMID: [Epub ahead of print] 33090858 PMC7899012

[ref32] Wylleman P. (2019). An organizational perspective on applied sport psychology in elite sport. Psychol. Sport Exerc. 42:89. 10.1016/j.psychsport.2019.01.008

[ref33] Wylleman P. Lavallee D. (2004). “A developmental perspective on transitions faced by athletes” in Developmental sport and exercise psychology: A lifespan perspective. ed. Weiss M. (Morgantown, WV: Fitness Information Technology), 507–527.

